# “Community health workers bring value and deserve to be valued too:” Key considerations in improving CHW career advancement opportunities

**DOI:** 10.3389/fpubh.2023.1036481

**Published:** 2023-03-08

**Authors:** Julie Smithwick, Jenesha Nance, Sarah Covington-Kolb, Ashley Rodriguez, Mike Young

**Affiliations:** ^1^Center for Community Health Alignment, The University of South Carolina Arnold School of Public Health, Columbia, SC, United States; ^2^Center for Applied Research and Evaluation, The University of South Carolina Arnold School of Public Health, Columbia, SC, United States; ^3^Baylor Scott & White Health, Dallas, TX, United States

**Keywords:** community health worker, workforce development, career pathway, COVID-19, professional development, health equity, leadership, promotores de salud

## Abstract

**Introduction:**

Community health workers (CHWs) are critical members of the public health workforce, who connect the individuals they serve with resources, advocate for communities facing health and racial inequities, and improve the quality of healthcare. However, there are typically limited professional and career building pathways for CHWs, which contribute to low wages and lack of career advancement, further resulting in turnover, attrition, and workforce instability.

**Methods:**

The Center for Community Health Alignment (CCHA), within the Arnold School of Public Health at the University of South Carolina, utilized a mixed-method data collection strategy to provide a more in-depth understanding of this issue and ways that employers, advocates, and CHWs can address it.

**Results:**

Themes across data sources emphasized the importance of retaining skilled and experienced CHWs and educating other health professions about CHWs' critical roles, and reported that doing so will result in decreased attrition professional growth, and improved program quality. CHWs and allies concluded that higher wages, valuing lived experience over formal education, and participation in additional training opportunities should be the primary factors considered for career advancement.

**Discussion:**

Utilizing input from experienced CHWs and CHW allies nationally, this article describes the importance of supporting CHW career advancement, shares best practices, and suggestions for designing strategies that organizations/employers can use to improve CHW career pathways to better support the CHW workforce and reduce attrition.

## 1. Introduction

There is growing recognition of how community health workers (CHWs) make significant impacts on the health and wellbeing of individuals and communities most affected by inequities (1–8). The American Public Health Association (APHA) definition of a CHW, endorsed by the National Association of CHWs (NACHW), is “a frontline public health worker who is a trusted member of and/or has an unusually close understanding of the community served. This trusting relationship enables the worker to serve as a liaison/link/intermediary between health/social services and the community to facilitate access to services and improve the quality and cultural competence of service delivery (9).”

Several systematic reviews and randomized control trials have found CHWs are associated with improved health outcomes in diabetes (10–13), multiple chronic diseases (14–16), cardiovascular risk reduction (17), hypertension (18), cancer screenings (19, 20), asthma (21), and mental health (22). CHWs help healthcare to accomplish the “Triple Aim” by contributing to improved care experiences and outcomes while maintaining cost-effectiveness (17, 19, 23–28). In addition, CHWs can contribute to enhancing the quality and cultural responsiveness of health and social services and support system-level changes that can have long-lasting impact (29).

While CHWs have been working in the United States for generations, during the recent 20 years, professional institutions have increasingly recognized their role and value in addressing health needs and gaps. In 2002, one of the Institute of Medicine's findings in their “Unequal Treatment” report was “Community Health Workers offer promise as a community-based resource to increase racial and ethnic minorities' access to health care and to serve as a liaison between healthcare providers and the communities they serve (2).” In 2010, the U.S. Department of Labor created a “Standard Occupational Classification” for CHWs, 21-1094. Furthermore, in 2010, the Patient Protection and Affordable Care Act mentioned CHWs 14 times, identified CHWs as a healthcare profession, and called for funding allocation for health promotion among underserved populations (30). During the COVID-19 pandemic, institutions such as the U.S. Centers for Disease Control and Surveillance (CDC) have funded and collaborated with CHWs to provide health outreach and education to hard-hit communities (31). Similarly, in 2022, the American Rescue Plan awarded over 220 million dollars toward community health worker training and capacity building nationwide (32).

As frontline public health workers during the COVID-19 pandemic, CHWs played important roles including communicating COVID-19 prevention and vaccination information to their communities in culturally responsive terms (33), as well as assisting in navigating the overwhelmed healthcare system and connecting clients to virtual medical care and mental health services (34). They also had a critical role in helping people address the myriad of social needs that the pandemic raised. As a result of the increased need in healthcare and public health spaces, coupled with increased evidence of effectiveness, the community health worker workforce is evolving rapidly in terms of professional identification and institutional recognition.

In practice, “CHW” is an umbrella term that encompasses dozens of titles, including peer health advisor, care navigator, outreach worker, community health representative, *promotores*, and more. This multitude of titles is one factor that has affected the potential consolidation of the role into a recognizable and definable profession (35). To address this, the CHW Core Consensus Project (C3 Project), building off the 1998 National Community Health Advisor Study, defined a set of core CHW roles and competencies in 2016 that CHW membership associations, public health institutions, and several states have endorsed (36).

The C3 Project and its predecessors identified CHWs' “connection to the community served” as their “most critical quality” (36). CHW employers prioritize this quality to identify and hire CHWs, more so than levels of formal education or years of employment experience. However, the latter forms the basis for how many institutions and organizations calculate salaries within their compensation and promotion structures. The result is that CHWs often have low salaries and status in the workplace and few opportunities for professional growth without leaving the profession.

The National CHW Common Indicators Project, a multi-institution national initiative to synthesize and improve CHW evaluation, highlights the importance of CHW workforce conditions and career advancement *via* two indicators. Indicator #1 is “CHWs' level of compensation, benefits, and promotion,” prioritizing this as an indicator of program quality. Indicator #12 is “Supportive and Reflective Supervision,” with CHWs recognizing this as, “...crucial factors affecting the ability of CHWs to grow as professionals, experience job satisfaction, and effectively promote health in their communities.” Considering that the improvement of the CHW work environment is reflected in two priority indicators for CHW success, pathways for advancement should be more clearly defined and incorporated into the workflow of organizations that employ CHWs.

The literature on the CHW workforce is growing, yet still somewhat limited. Global literature identifies high attrition rates for CHWs, with contributing factors including low and inconsistent salaries, lack of support, and leaving for better positions (37–45). In a systematic review, Kok et al. found professional growth as a motivating factor for CHWs, and a lack of career advancement options as a demotivating one (46).

In the United States, CHW attrition rates can also be high, with low salaries, low professional status, and lack of opportunities for professional growth driving dissatisfaction (47, 48). Farrar et al. (48) found that addressing barriers to education, training, and promotion led to an improvement in job satisfaction for CHWs. Anabui et al. (47) found that despite the challenges faced in the workplace, most CHWs want to retain their identities, rather than move to other health or helping professions for advancement. They found that clear criteria and opportunities for promotion within their field, including incentives that recognize lived experiences, were motivating for CHWs.

While NACHW, APHA, and the C3 Project have defined CHWs' roles, skills, and qualities, and many state CHW associations have established core competency training and certifications, there is minimal standardized guidance around best practices for hiring or promoting CHWs. Existing guidance emerged from the 1998 National Community Health Advisor (NCHA) Study, which recommended 18 qualities that employers should seek when hiring CHWs. This guidance, although not commonly adopted, could be built upon and shared to promote the enhancement of the CHW workforce. Opportunities for advancement are particularly critical for CHWs, many of whom are themselves from (or closely tied to) populations experiencing inequities, such as members of racial minority groups, immigrants, people with low income, people who were formerly incarcerated, or people in recovery. Lack of mobility may perpetuate their economic and social vulnerabilities.

The purpose of this project was to gather and synthesize first-hand perspectives from CHWs and their allies on this workforce challenge. By collecting CHW input from multiple sources, we describe why it is important to support CHW career advancement, and what employers and advocates can do to develop CHW career tiers or advancement strategies and reduce the threat of losing this vital workforce at a time when the need for their work and expertise has become even more evident.

## 2. Methods

With support from Johnson & Johnson, in line with their Our Race to Health Equity initiative (49), the Center for Community Health Alignment's Community Health Worker Institute (CHWI) and Center for Applied Research and Evaluation (CARE) at the University of South Carolina Arnold School of Public Health (ASPH) gathered insight from four sources for this project: qualitative data from the South Carolina CHW Ambassadors and CHW Best Practices Council; the National Association of CHWs' (NACHW) 2021 Annual CHW Workforce Survey, a rapid-feedback session of CHWs at the NACHW Unity Conference; and qualitative interviews with six CHW managers or supervisors. The Institutional Review Board at the University of South Carolina approved the study.

### 2.1. The South Carolina CHW ambassadors and CHW best practices council

A foundational component of CHWI is its engagement of a team of “CHW Ambassadors,” experienced CHWs representing a variety of races, ethnicities, geographical locations, populations served, and organizational affiliations. In 2019, the CHWI team selected 10 applicants to be the first cohort of CHW Ambassadors. They contributed to developing plans and strategies for CHW training, capacity building, data collection, and other critical components of CHWI's mission. In addition to the Ambassadors, CHWI invited seven CHW managers or supervisors to be part of a CHW Best Practices Experts Council, whose goal was to determine best practices for implementing and developing CHW programs and growing the CHW workforce in South Carolina effectively. The input from the Best Practices Expert Council on the need for CHW career advancement and a tiered certification structure was the primary impetus for this study. For a year, the council met six times to discuss job descriptions and the factors that should merit increased benefits, recognition, pay, and leadership opportunities for CHWs. The Council discussed various viewpoints until a consensus was reached.

### 2.2. NACHW's 2021 National CHW workforce survey

The National Association of CHWs (NACHW) invited key influencers, funders, and CHWs leaders to join workgroup sessions toward developing this survey. The workgroup conducted extensive literature review searches, cross-referenced keywords, and identified knowledge gaps regarding the profession. CHWI collaborated with NACHW and its team of CHW advisors to add three additional questions regarding career advancement to the 51-question survey. The survey launched in June 2021, and collected responses through September 2021, ultimately compiling 867 completed surveys (772 in English and 95 in Spanish).

### 2.3. A rapid feedback session at the 2021 NACHW annual Unity Conference

In July 2021, 160 CHWs and CHW allies attended an invited session at the virtual NACHW Unity Conference, titled “CHWs' Career advancement strategies: Nothing about us without us.” Two CHW leaders from the Center for Community Health Alignment conducted the session, which was in English. The facilitators posed open-ended questions about CHW career pathways and collected responses *via* Mentimeter, an online polling platform. In total, 80 individuals responded to four questions posed in a rapid-polling activity. Among attendees that responded to the poll, 70 (87%) identified as CHWs, and 10 (13%) as CHW allies (a supporter, an employer, or a researcher of CHWs). Of CHWs, 35% had been a CHW for more than 5 years, 18% for < 1 year, and 36% between 1 and 4 years. Most of the CHWs and allies worked in a healthcare setting (34%) or a community-based organization (33%). Others worked in a public health department (9%), university (9%), or CHW organization or association (8%). Participants did not divulge their geographical locations. The questions were either open-ended or multiple choice and were collected and organized by themes using a deductive approach.

### 2.4. Individual interviews

Members of the research team conducted six semi-structured interviews with CHWs that are managers or supervisors of other CHWs. The interview guide consisted of several open-ended questions about the CHW career paths at their organizations. Topics included CHW compensation, promotion opportunities, and ways to support future CHWs in career progression. Four CHW managers were from South Carolina organizations, with another from Chicago, Illinois, and one from Dallas, Texas. Three managers worked in community-based settings, while the others worked in healthcare settings. Interviews were conducted *via* the Zoom telecommunication platform. CHWI recruited interviewees based on their knowledge of the CHW profession and experience managing CHW teams. To protect participant privacy, recordings, transcriptions, and analyses were de-identified and stored on a private cloud-based drive. In recognition of their content expertise, participants received a stipend for their participation. The interviews were transcribed using Otter.ai software.

To analyze data, information was coded and analyzed for themes using Microsoft Excel (50, 51). The study team used the constant comparative method (52). As a group, we read through each transcript, noted emergent themes, and compared themes identified in previous transcripts. Furthermore, the study team discussed various viewpoints between team members until a consensus was reached.

## 3. Results

Several themes emerged across the data sources regarding the need to advance the CHW workforce (the “why”) including the importance of retaining skilled CHWs, building respect and appreciation for the profession, and professional growth that will improve program and organizational quality. The themes about the development of opportunities for CHW advancement (the “how”) included the importance of salary improvement, making advancement decisions based on lived experience more than formal education, and providing opportunities for additional CHW training and professional development. The responses to the NACHW workforce survey are listed in Tables 1, 2, and Unity Conference session feedback is in Tables 3–5.

**Table 1:** Top options that CHWs believe should be incorporated into a CHW career path.

**Response**	**English *n* = 772**	**Spanish *n* = 95**	**Total *n* = 867**
Salary increases	621 (80.4%)	45 (47.4%)	666 (76.8%)
New project/program development	371 (48.1%)	68 (71.6%)	439 (50.6%)
Retention of the CHW title with advancement	398 (51.6%)	36 (37.9%)	434 (50.1%)
Supervisory roles	313 (40.5%)	28 (29.5%)	341 (39.3%)
Authority to lead project teams	255 (33.0%)	46 (48.4%)	301 (34.7%)
Training of non-CHWs	222 (28.8%)	32 (33.7%)	254 (29.3%)

Participants were allowed to select multiple responses.

Source: NACHW Annual Workforce Survey, 2021.

**Table 2:** The top factors that CHWs believe should be valued for a CHW to advance in their career.

**Response**	**English *n* = 772**	**Spanish *n* = 95**	**Total *n* = 867**
Additional CHW training (continuing education, specialties)	548 (71.0%)	63 (66.3%)	611 (70.5%)
Completing CHW certification	432 (56.0%)	55 (57.9%)	487 (56.2%)
Experience mentoring or training other CHWs	415 (53.8%)	44 (46.3%)	459 (52.9%)
Years providing CHW services	372 (48.2%)	47 (49.5%)	419 (48.3%)
Community member evaluation and feedback	246 (31.9%)	36 (37.9%)	282 (32.5%)
Formal education	183 (23.7%)	20 (21.1%)	203 (23.4%)

Participants were allowed to select multiple responses.

Source: NACHW Annual Workforce Survey, 2021.

**Table 3:** Most frequently appearing codes for responses to open-ended question, “Why is more attention needed on CHW career advancement options?”

**Response**	**#**
Respect for the profession	22
Better care for the community	20
CHW retention	17
CHW income	17
Lack of understanding of the CHW role	14
CHWs' unique qualities	10

Source: responses at rapid feedback session during the 2021 NACHW Unity Conference.

**Table 4:** Benefits CHWs believe should be available as they advance in their careers, in descending order.

**Response**	**#**	***n* = 75**
More pay	68	90.7%
More professional development	65	86.7%
Management opportunities	47	62.7%
Opportunity to supervise	43	57.3%
Freedom to operate more independently	37	49.3%
Speaking opportunities	36	48.0%
More responsibilities	25	33.3%

Source: survey distributed at rapid feedback session during the 2021 NACHW Unity Conference.

**Table 5:** Most frequently appearing codes for responses to open-ended question, “What factors need to be considered to move CHWs from one level to the next?”

**Response**	**#**
Experience	27
Training	24
Education	20
Lived experience/community connection	18
Organizational support	8

Source: responses at rapid feedback session during the 2021 NACHW Unity Conference.

### 3.1. CHW advancement is critical: “*The why”*

#### 3.1.1. Retention

Studies about CHW attrition found that a lack of opportunities for professional growth contributes to turnover rates (47, 48). This is reflected in our findings, first emerging from the CHW Best Practices Council, which identified that the lack of advancement opportunities caused many CHWs to leave the field. They advocated that employers of CHWs and the South Carolina CHW Credentialing Council make CHW retention a high priority by developing tiered levels of certification.

Participants at the Unity Conference agreed, “Having more options for career advancement generally means more people staying in the field, only becoming better at what they do, with the pay they deserve, helping as much people as they can.” Another said, “Because the way things are now, we are losing strong CHWs from the field. Those that are most connected to the community are now removed which causes a loss of trust.”

#### 3.1.2. Respect for the CHW position

The relationship between CHW advancement and a perceived lack of respect from other professionals emerged at the Unity Conference (Table 3). One CHW stated, “We are treated like housekeeping... a necessary position that is undervalued.” Another respondent said, “We are living in poverty, have skills and experience and education, but are trapped, underpaid and underappreciated with no hope of improvement; we are dedicated missionaries.” Another wrote, “Because for many companies [the CHW profession] is an unknown field and they do not understand how valuable CHWs are and don't know what to do with us.” Yet another stated, “I think people believe it's just volunteer work.”

The interviewed managers agreed. One manager stated, “Yeah, I just think the... [profession]... is brand new. Like, we are learning about all the benefits that our work offers to the community. … We need to create awareness to the funders about the value that we provide to the community and to the medical services and the government, how much money they saved… I think like doing research, like a study of the return on investment… real numbers that you can show to the funders, so they can see the benefit of our profession.” Another suggested that awareness building is an important role for CHW allies, “Continue to work and push work with organizations, to develop Community Health Worker programs, and help them to understand the role of Community Health Workers and why they're important, continue to advocate for Community Health Worker pay.” Educating other professionals and team members about the role of CHWs will help showcase their value in the workplace, further incentivizing them to stay and grow.

#### 3.1.3. Professional growth and quality improvement

The interviewees also mentioned that CHWs' professional growth will ultimately result in improved services to patients and participants. “We all want to get better all the time and, and make more money, and just think that you are growing, that you are learning, and that you are growing in your profession. Just like it is good for us and it motivates you to have like a better position, a better salary. I think that's important for everybody.” Another interviewee stated, “Oh, it gives them something to strive for, it gives them something to look forward to, you know, I'm saying it allows them to take the initiative to improve themselves, in professional development.”

### 3.2. Factors to consider when developing CHW advancement pathways: “*The how”*

#### 3.2.1. Salary

Across data sources, CHWs identified “salary increases” as the most important factor to consider in advancement opportunities. “Higher salary” was the most frequently mentioned priority for CHWs in both the NACHW Workforce Survey (Table 1) and the Unity Conference session (Table 4) when asked what should be incorporated into advancement pathways. One conference attendee commented, “CHWs are often one paycheck away from needing the services we provide.”

The CHW managers are aware of the impact of low salaries on their CHWs; one said, “It's just I have been advocating for my team to get a better salary, but it is very hard for us to get the resources. … I really would like to have more for my Community Health Workers because they work so hard. And they really deserve to have a better salary.” However, they also deal with the challenges of finding funding sources for those salaries. One manager said, “You know how it works when you work with grants, you have this person for 1 year and she learned so many things, she's gained so many skills, and then she has to leave because I don't have any more funds.” Two CHW managers suggested institutional funds or insurance payments be aligned with CHW services. One suggested, “Oh, that's the biggest challenge for me that I would really like... maybe the hospitals to take over these programs and say, ‘okay, I'm going to pay you like, to permanently to do the work.”' Another mentioned, “[A] patient came in, and we assisted them with food insecurity, boom, that should be a code. Right? That should be a compensation point.”

#### 3.2.2. Valuing lived experience above formal education

At the Unity conference session, many responses to the question, “What factors should be considered to move CHWs from one level to the next?” were related to experience as CHWs and lived experience as a member of the communities served (Table 5). Answers to the question included, “Respecting experience and not just the letters after your name,” and “Experience in the field and the resources we bring... how we are respected in the community.” One CHW said, “Because CHW's speak in everyday people's language! That's why what we do works!”

Multiple interviewed CHW managers agreed they would hire or promote other CHWs based on lived experience. One manager stated, “So I struggle with that sometimes, because I sometimes do get caught in okay, what kind of education do they have? … But then I had to, I had to catch myself sometimes. That's not what is at the heart of a Community Health Worker. It's the community. It's the person. Its, you know, what role have they played in their community?” When asked what factors are important in promoting CHWs, another CHW manager stated that, “experience dealing with the population that we're trying to target… and being able to meet those patients where they're at,” was important. Another manager said, “I found out some of the ones, you know, that that didn't have that that degree or, or may not always have had a medical experience are the best ones.”

The value of lived experience and community connections contrasts with many employing institutions' emphasis on formal education and credentials. In the NACHW workforce survey, when asked about barriers to their success as CHWs, 25% of CHWs identified higher-level education and 21% “other credentials” (53). CHWs have deep knowledge and experience in the community; determining how to place value on this quality in an institutional setting is critical.

#### 3.2.3. CHW training and professional development

While most respondents did not prioritize associating CHW advancement with formal education, there is a theme of wanting CHW training, professional development, and certification as means of advancing. The CHW Best Practices Council recommended that additional CHW-focused education and experience should help move a CHW from one tier to the next.

The national survey respondents agreed; CHWs ranked “additional CHW training” as the top factor to be valued in their career paths (Table 2). This was followed by CHW certification, then experience mentoring other CHWs. An interviewee also agreed, suggesting training as a method of supporting CHW advancement, “I would say continue to provide educational pathways for Community Health Workers, professional development.” Another interviewee suggested that organizations should, “…assess company needs and provide training and education.”

## 4. Discussion

These findings show that more effective and equitable career pathways for CHWs would provide motivation for CHWs to remain in the workforce, promote retention, recognize effective work and leadership, and contribute to organizational quality improvement. CHW career advancement pathways should recognize the unique contributions of CHWs to health improvement, take into consideration the best practices offered by the workforce, and support CHWs in addressing the challenges and barriers that exist.

In the past few years, a growing number of institutions and organizations have realized the need to hire and integrate CHWs into their work. However, the CHW field faces multiple threats that can result in attrition and burnout and put the workforce and employers at risk of not being able to meet the growing demand. CHWs have reported feeling undervalued, underpaid, and not respected. By not providing opportunities for CHWs to grow in their field, organizations risk losing those CHWs with the most experience, as opposed to building on that expertise to support newer CHWs.

### 4.1. Challenges to be addressed

The barrier that was most frequently mentioned by CHWs and CHW allies was funding, both in terms of having enough funding to offer higher salaries and having sustainable funding so that positions are not dependent on grants and other one-time funding sources. Due to the value of CHWs' lived experience and connection to the populations they serve, CHWs can be at high risk of poverty, which makes offering livable wages essential. In addition, time limited and insufficient compensation pose substantial risks to the CHW workforce. Our findings suggest that having more sustainable and equitable funding will facilitate the retention of CHWs.

Another major challenge pertains to human resource systems and processes placing value on higher education and formal degrees rather than lived experience. Foremost, higher education can be financially cumbersome for CHWs, many of whom hail from marginalized populations. Similarly, CHW managers, CHWs, and the C3 Project agree that lived experience is a core quality of a CHW. Therefore, loosening requirements surrounding formal education and valuing lived experience can assist in finding and promoting the right individuals for CHW positions. It can also assist in building financial equity for CHWs because they will have opportunities for advancement without incurring additional debt. Further emphasis needs to be placed on understanding and dismantling systemic racism within organizations due to the part that it plays in maintaining hierarchical leadership that keeps those with privilege in power. The role of privilege and power dynamics needs to be recognized and addressed in order to determine paths to leadership that are more equitable, open, and transparent.

Finally, there is a need for more awareness of CHW's roles and contributions to public health and healthcare. Many stated that employers such as hospitals and state or federal agencies lack awareness of the roles, qualities, and importance of CHWs. Similarly, community members may not understand the role of a CHW. Developing strong communication plans that include training employers and marketing to the public can assist in raising awareness of the CHW profession, creating more respect for the CHW role and scope, helping keep experienced CHWs in the workforce, and incentivizing others to join the field.

### 4.2. Designing CHW advancement pathways

Almost all CHWs and allies reported that most CHWs want to stay in and be able to progress within their field, as opposed to having to leave the CHW workforce to pursue additional opportunities. However, many CHWs stated that employers will not allow them to progress without adopting a new title or professional identity. Developing a tiered CHW advancement framework, such as the ones in the case examples included, can guide employers and advocates in their efforts to promote and retain CHWs. Advancement factors can include additional training, professional development, certifications, mentorship of other CHWs, years in the CHW field, and the quality of their work. CHWs strongly favor their profession's own training, certification, and experience as factors for advancement, more than formal education or external qualifications. Upon advancing, CHWs should be granted opportunities to participate in leadership roles, mentorship, program design, and advocacy.

### 4.3. Examples

Later are two examples of organizations that have created and implemented CHW career advancement pathways, with strong and intentional input from CHWs.

#### 4.3.1. CHW tiered system at Baylor Scott & White Health

Baylor Scott & White Health (BSWH), the largest not-for-profit healthcare system in Texas and one of the largest in the United States, employs CHWs to help patients navigate an increasingly complex healthcare system, facilitate self-management of chronic diseases, and connect patients to primary care medical homes. BSWH CHWs act as peers, navigators, advocates, educators, and promoters of improving outcomes and quality of life for the patients they serve.

In 2007, BSWH hired one CHW as a diabetes educator. In 2009, the team expanded to four CHWs through the Diabetes Equity Project, and in 2011, BSWH created new positions, marking the start of BSWH's current CHW career ladder efforts. Between 2011 and 2014, the integration of CHWs expanded even more through various grant and funding opportunities.

As the system developed more CHW programs, CHW supervisors recognized the need for a systemic approach to CHW support that could bridge the programs. They created the CHW Development Council to meet this need. This Council utilized key data derived from annual workforce feedback surveys at the BSWH CHW Summit and the Texas core competencies for public health professionals to build a career ladder. Rather than encouraging BSWH CHWs to pursue growth through other career ladders such as becoming social workers or other healthcare professionals, they determined it was more effective and supportive to build a CHW career ladder that respects the CHWs' passion and path, thus allowing BSWH to retain talented and invaluable CHWs at all levels for the last decade.

The CHW Development Council developed the advancement levels in Table 6 and provided the levels to supervisors as a framework they can use to help grow and retain their CHWs. Currently, BSWH employs over 120 CHWs.

**Table 6:** Baylor Scott & White Health CHW positions.

**Position**	**General description**
CHW in-training	Embedded community member that has the desired experience and knowledge of their community but does not necessarily have the required Texas Department of Health Services CHW certification; they are required to complete certification training within 1 year of employment.
CHW I	Experienced CHW, with current DSHS certification
CHW II	Experienced CHW, with current DSHS certification, that may have taken a team lead role and/or have applicable experience
CHW supervisor	Experienced, “veteran” CHW, with current certification, who has the capacity to manage a team
CHW manager	Experienced CHW that supports multiple care settings, may directly oversee CHW staff, and may serve at a system level in a project management capacity to support and drive CHW initiatives

#### 4.3.2. South Carolina CHW tiers

As previously mentioned, the South Carolina CHW Best Practices Council (BPC) identified CHW career advancement as a high priority for the CHW workforce in South Carolina. The South Carolina CHW Credentialing Council (SCCHWCC), which is supported by the South Carolina Community Health Worker Association, is the statewide approving body for CHW training programs, certification, and continuing education. The SCCHWCC is composed of at least 51% CHWs, along with representatives from the state Medicaid institution, the state public health department, AHEC, higher education, and a health insurance entity.

The BPC worked to draft examples of tiered CHW job descriptions, based on the different requirements for the position. The Council also recommended that, statewide, there should be multiple tiers of CHW certification, and that additional CHW-focused education and experience should be what help move a CHW from one tier to the next.

Based on the work initiated by the BPC, CHWCC made enhancements to the CHW credentialing process, creating a three-tiered system for certified CHWs. The Credentialing Council drafted these tiers and reviewed them multiple times with the BPC. In April 2022, the three-tiered certification in Table 7 was approved and it was launched in January 2023. In the future, efforts will be made to educate employers on the certification tiers, to encourage them to adopt them, along with appropriate advancement in salaries and opportunities for CHWs.

**Table 7:** South Carolina CHW tiers.

**CHW position**	**General description**
Certified community health worker (CCHW)	• Successful completion of a SCCHWCC approved CHW Core Competency Curriculum: 80 h classroom, 80 h practicum, SCCHW Examination • Registering on the South Carolina Community Health Worker Association (SCCHWA) CHW Portal
Certified community health worker II (CCHW II)	• Minimum of 4 years working the field • Bi-Annual Recertification (24 h every 2 years) • Certification of Completion of a minimum of 4 CHW Specialty Tracks • Demonstration of at least 1 year in CHW leadership (CHW ambassador, SCCHWA board, active involvement in SCCHWA workgroup, active involvement in CHW regional or national committee or initiative, CHW Preceptor, CHW supervisor, CHW program leadership or program development at organizational level) • Updated profile on the SCCHWA CHW Portal • Application submission
Certified community health worker III (CCHW III)	• Minimum of 8 years working the field • Bi-Annual Recertification (24 h every 2 years) • Certification of Completion of a minimum of 6 CHW Specialty Tracks • Demonstration of at least 2 years in CHW leadership (CHW ambassador, SCCHWA board, active involvement in SCCHWA workgroup, active involvement in CHW regional or national committee or initiative, CHW Preceptor, CHW supervisor, CHW program leadership or program development at organizational level) • Updated profile on the SCCHWA CHW Portal • Application submission

### 4.4. Limitations

Limitations of this study include a lack of consistency across data sources; the questions were similar, but not identical. To address this, the team gathered data from multiple sources to make sure the themes were accurate and consistent.

Respondents of the national CHW survey may have been impacted by survey fatigue bias, due to the length of the questionnaire. In addition, the interviews and Unity Conference sessions were in English. Aligning interview data of Spanish speakers with the survey data could have brought more insight to researchers around the differences between how English and Spanish-speaking CHWs are employed and promoted. Further research can be conducted in various languages and with CHWs from different backgrounds to determine if there are nuances in factors affecting CHW advancement.

## Data availability statement

The original contributions presented in the study are included in the article/supplementary material, further inquiries can be directed to the corresponding author.

## Ethics statement

The studies involving human participants were reviewed and approved by University of South Carolina Institutional Review Board. The NACHW survey and conference poll were anonymous, and did not require written consent. Individual interview participants provided their verbal consent to participate in this study.

## Author contributions

JS and MY designed the study and led data collection. JN and SC-K contributed to analyzing the data and preparing the manuscript. AR reviewed for relevancy to the field and provided case study input. All authors contributed to the article and approved the submitted version.

## Appendix

### CHW interview guide

Do I have permission to record this interview?

Thank you for taking the time to interview with me today. First, I'm going to get some information about you and your role within your organization.

1. Tell me a little about yourself and your work at your organization.

a. How long have you been a CHW? How long have you worked at your organization?

b. About how many CHWs does your organization employ?

c. How many do you directly supervise?

d. What are the funding sources for your CHWs pay?

2. Next, we're going to discuss the career path for CHWs in your organization. When I say career path, I mean how people grow or advance in their job, such as opportunities for promotions or pay raises. Does your organization have a career ladder for CHWs? If so, tell me about them.

a. Does your organization have education or experience requirements to promote CHWs?

b. What barriers or challenges have you faced when developing a career ladder? How did you address these barriers?

c. Why do you think having a career ladder is important?

d. What factors should be considered to move CHWs from 1 level to the next?

e. What qualities/personality traits do you look for when hiring or promoting a CHW?

3. What does compensation look like for CHWs at your organization? What is the lowest and highest salary range?

a. What types of benefits do your CHWs receive?

b. Any other benefits (mileage, per diem, etc)

c. What about the CHW model at your organization do you think is unique?

4. If you had the opportunity to change anything about the CHW profession, what would it be?

a. Have you heard anything from former CHWs about why they left the position?

5. In your opinion, how can we better support CHWs in advancing their careers?

6. What else can you share with me that could be helpful as we try to work on this?
